# Long-Term Neurotoxicity of Chemotherapy in Adolescents and Young Adults Treated for Bone and Soft Tissue Sarcomas

**DOI:** 10.1080/13577149878055

**Published:** 1998-06

**Authors:** Helena M. Earl, Sean Connolly, Christos Latoufis, Karen Eagle, Catherine M. Ash, Clare Fowler, Robert L. Souhami

**Affiliations:** 1 Department of Oncology University of Cambridge School of Clinical Medicine Addenbrooke's Hospital Box 193, Hills Road Cambridge CB2 2QQ UK; 2 Department of Clinical Neurophysiology Middlesex Hospital University College London Medical School London UK; 3 Department of Oncology University College London Medical School London UK

## Abstract

*Purpose.* To study the long-term neurotoxicity of chemotherapy in adolescents and young adults treated for bone and soft tissue sarcomas.

*Patients and Methods.* Thirty-six adolescents and young adults (median age 17 years) were examined following chemotherapy for bone and soft tissue sarcomas. Twenty-nine (29/36) had received cisplatin (median 400 mg/m^2^), 15/36 ifosfamide (median 20 g/m^2^), and 12/36 vincristine (median 16 mg). Neurotoxicity was assessed at a median of 8 months (range, 1–54 months) after completion of chemotherapy by clinical examination, nerve conduction studies, audiograms and autonomic function tests. The same nerve conduction studies were carried out in 20 normal volunteers to define normal ranges in this age group.

*Results.* Sixteen patients (44%) had a significant reduction in deep tendon reflexes, and this clinical parameter correlated well with abnormalities detected in nerve conduction studies. Vibration perception threshold (VPT) was raised in 20/36 patients (55%) and this was the most sensitive single test in the assessment of neuropathy. There was a significant correlation between VPT and cumulative cisplatin dose received in mg/m^-2^ (*r*=0.607, *p*<0.01). Ten of 29 patients (35%) had abnormal nerve conduction studies with a pattern characteristic of sensory axonal neuropathy. No patient complained of auditory symptoms, but minor high tone hearing loss was detected by audiograms in 5/28 patients who had received cisplatin. No patients had symptoms of autonomic neuropathy, but autonomic function tests showed minor abnormalities in 4/22 patients tested, and all had received cisplatin.

*Conclusions.* This study demonstrates significant, although asymptomatic, long-term neurotoxicity of cisplatin in adolescents and young adults receiving chemotherapy for bone and soft tissue sarcomas. Follow-up studies are planned to assess whether these neurological deficits improve with time.


*Sarcoma* (1998) **2**, 97–105

ORIGINAL ARTICLE

### Long-term neurotoxicity of chemotherapy in adolescents and young adults treated for bone and soft tissue sarcomas

HELENA M. EARL,[1] SEAN CONNOLLY,[2] CHRISTOS LATOUFIS,[3] KAREN EAGLE,[3] CATHERINE M. ASH,[3] CLARE FOWLER[2] & ROBERT L. SOUHAMI[3]

[1]*Department of Oncology, University of Cambridge School of Clinical Medicine, Cambridge &* [2]*Department of Clinical Neurophysiology, Middlesex Hospital, University College, London Medical School, London &* [3]*Department of Oncology, University College London Medical School, London, UK*

**Abstract**

*Purpose.* To study the long-term neurotoxicity of chemotherapy in adolescents and young adults treated for bone and soft tissue sarcomas.

*Patients and Methods.* Thirty-six adolescents and young adults (median age 17 years) were examined following chemotherapy for bone and soft tissue sarcomas. Twenty-nine (29/36) had received cisplatin (median 400 mg/m$^2$), 15/36 ifosfamide (median 20 g/m$^2$), and 12/36 vincristine (median 16 mg). Neurotoxicity was assessed at a median of 8 months (range, 1–54 months) after completion of chemotherapy by clinical examination, nerve conduction studies, audiograms and autonomic function tests. The same nerve conduction studies were carried out in 20 normal volunteers to define normal ranges in this age group.

*Results.* Sixteen patients (44%) had a significant reduction in deep tendon reflexes, and this clinical parameter correlated well with abnormalities detected in nerve conduction studies. Vibration perception threshold (VPT) was raised in 20/36 patients (55%) and this was the most sensitive single test in the assessment of neuropathy. There was a significant correlation between VPT and cumulative cisplatin dose received in mg/m$^2$ ($r = 0.607$, $p < 0.01$). Ten of 29 patients (35%) had abnormal nerve conduction studies with a pattern characteristic of sensory axonal neuropathy. No patient complained of auditory symptoms, but minor high tone hearing loss was detected by audiograms in 5/28 patients who had received cisplatin. No patients had symptoms of autonomic neuropathy, but autonomic function tests showed minor abnormalities in 4/22 patients tested, and all had received cisplatin.

*Conclusions.* This study demonstrates significant, although asymptomatic, long-term neurotoxicity of cisplatin in adolescents and young adults receiving chemotherapy for bone and soft tissue sarcomas. Follow-up studies are planned to assess whether these neurological deficits improve with time.

**Key Words**: *Adolescent cancer, long-term cisplatin neurotoxicity, bone and soft tissue sarcomas.*

### Introduction

Intensive combination chemotherapy has greatly improved the prognosis of childhood and adolescent tumours. In osteosarcomas, for example, the cure rates for UK children under the age of 16 years have risen from 20% in the mid-1970s, to 60% at the present time, an improvement attributable to intensive adjuvant chemotherapy.[1] Similar advances are now taking place in the treatment of Ewing's sarcoma.[2] This is a considerable triumph for modern cancer management. The long-term adverse effects of cancer treatments in adolescents are of considerable importance since, each year in the UK, 1200 young adults will have a history of having been cured of a cancer in childhood or adolescence. If their life span is normal this will represent over 50 000 UK adults by the year 2030.

There is little published data on long-term neurotoxicity of chemotherapy in the adolescent age group. Indeed, the normal ranges in adolescents are not well established for many of the available tests of neurological function. Previous studies have not always stated what the reference population has been.[3,4] Although the reported neurotoxicity has not, in the main, been clinically severe, it is important nonetheless to establish the frequency and severity of sub-clinical neurotoxicity. Chemotherapy-induced nerve damage may have important implications for the future, because other potentially

Correspondence to: H. Earl, Department of Oncology, University of Cambridge School of Clinical Medicine, Addenbrooke's Hospital, Box 193, Hills Road Cambridge CB2 2QQ, UK. Tel: 01223 274312; Fax: 01223 412213; E-mail: hme22@cam.ac.uk

1357-714X/98/020097-09 ©1998 Carfax Publishing Ltd neurotoxic compounds may have an additive neurotoxic effect in these patients. In later life, common illnesses such as diabetes may produce more severe neuropathy because the starting point is not one of a normal peripheral nervous system.

The aim of the present study was to establish the frequency and severity of long-term sub-clinical neurotoxicity from cytotoxics currently used in adolescents and young adults with bone and soft tissue sarcomas, compared with a reference population of normal volunteers of similar age, using the same methodology.

### Patients and chemotherapy protocols

Thirty-six patients with bone and soft tissue sarcomas were studied. Patients' characteristics are shown in Table 1. There were 19 males and 17 females, with a median age at diagnosis of 17 years (range, 3–29 years), and a median age at assessment of 19 years (range, 8–30 years). Patients were assessed at a median of 8 months from the completion of chemotherapy (range, 1–54 months). Twenty-one patients were between 1 and 12 months, eight patients between 13 and 24 months, and five patients between 25 and 54 months from the completion of chemotherapy. Diagnoses were as follows: osteosarcoma (28), Ewing's sarcoma of bone (five), soft tissue sarcoma (two), and spindle cell sarcoma of bone (one). All patients were treated on National and International protocols which included: (1) MRC osteosarcoma protocol BO03, a randomised study of cisplatin/doxorubicin versus multidrug adjuvant chemotherapy;[5,6] (2) MRC/EOI (European Osteosarcoma Intergroup) protocol for metastatic or axial skeleton osteosarcoma; (3) UKCCSG (United Kingdom Children's Cancer Study Group) protocol for Ewing's sarcoma of bone; (4) MRC pilot study in osteosarcoma of cisplatin, doxorubicin, ifosfamide and high-dose methotrexate; (5) phase 2 study of methotrexate, ifosfamide and doxorubicin in malignant fibrous histiocytoma of bone.[7] The protocols, chemotherapy and duration of treatment are shown in Table 2, and the actual doses of chemotherapy received are shown in Table 3.

**Table 1.** *Patient characteristics*

| Diagnosis | Number of patients |
|---|---|
| Osteosarcoma | 28 |
| Ewing's sarcoma | 5 |
| Soft tissue sarcoma | 2 |
| Spindle cell sarcoma of bone | 1 |

*Other characteristics* (median (range)) : age at diagnosis (years) 17 (3–29); age at assessment (years) 19 (8–30); time from the end of chemotherapy (months) 8 (1–54).

### Methods

### *Clinical examination*

All patients had full clinical neurological assessment and nerve conduction studies. Twenty-eight patients had bilateral audiograms, and 22 patients had assessment of autonomic function. Clinical neurological history included detailed questions concerning peripheral sensory and motor neuropathy, unsteadiness, Lhermitte's syndrome, Raynaud's phenomena.[8,9] auditory symptoms and symptoms of autonomic neuropathy. Symptoms were assessed as either acute (i.e., immediate, during each chemotherapy course), intermediate (i.e., during the 6–12 months of chemotherapy), or as late effects (after completion of all chemotherapy). Clinical neurological examination was carried out by the same investigator (HME) with full neurological assessment of motor and sensory systems, including joint position sense and vibration sense (tuning fork 128 c/s). Deep tendon reflexes (DTRs) were assessed and scored as normal (score = 1), reduced (only present with reinforcement, score = 0.5) or absent (score = 0). Biceps, triceps, supinator, knee and ankle DTRs were scored to give a maximum cumulative normal score of 10. Reflex scores of 6 or less were considered abnormal.

### *Nerve conduction studies (NCS)*

Neurophysiological assessment of all patients was carried out by the same doctor (SC), who also examined 20 healthy volunteers (14 females and six males) ranging in age from 18 to 26 years (mean age = 22 years). Full neurophysiological investigations included nerve conduction studies and the measurement of vibration perception and thermal thresholds. Lower limb nerve conduction studies were performed using surface electrodes and recorded on a Medelec MS 92 electromyograph. Recordings were made of sural and medial plantar sensory nerve action potential (SNAP) latency and amplitude, and sensory conduction velocities (SCV) were calculated for these nerves. The compound muscle action potential (CMAP) amplitude and latency from flexor hallucis brevis after supra-maximal stimulation of the posterior tibial nerve at the ankle and the knee was recorded, and the motor conduction velocity (MCV) for the nerve calculated. Record was also made of a minimum of 10 f-wave latencies from flexor hallucis brevis while giving supra-maximal stimuli to the posterior tibial nerve at the ankle.

The vibration perception threshold (VPT) was measured using a Biothesiometer. This instrument has a dial which reads the voltage applied to the probe causing it to vibrate. The movement of the probe (in $\mu$M) is linearly related to the square of the voltage. With the subject in a semi-recumbent position, the instrument was placed vertically on the

**Table 2**. *Adjuvant chemotherapy protocols*

| No. of patients | Diagnosis | Protocol | Drugs | Intended duration (weeks) |
|---|---|---|---|---|
| 22 | Osteosarcoma | MRC BO03<br>Short arm<br>vs. | Cisplatin<br>Doxorubicin | 22 |
| | | Long arm | Cisplatin<br>Doxorubicin<br>Methotrexate<br>Vincristine<br>Bleomycin<br>Cyclophosphamide<br>Actinomycin | 44 |
| 5 | Osteosarcoma | MRC/EOI/PIA BO04 | Cisplatin<br>Ifosfamide<br>Doxorubicin | 22 |
| 5 | Ewing's Sarcoma | UKCCSG IVAD | Ifosfamide<br>Doxorubicin<br>Actinomycin<br>Vincristine | 52 |
| 2 | Soft tissue sarcoma | Off study IVA | Ifosfamide<br>Doxorubicin<br>Vincristine | 18 |
| 1 | MFH of bone | Phase II Study | Ifosfamide<br>Doxorubicin<br>Methotrexate | 16 |
| 1 | Osteosarcoma | MRC Pilot | Ifosfamide<br>Cisplatin<br>Doxorubicin | 22 |

MFH, malignant fibrous histiocytoma; UKCCSG, United Kingdom Childhood Cancer Study Group; EOI, European Osteosarcoma Intergroup Study.

**Table 3**. *Total doses of chemotherapy received*

| | | Total dose (mg/m$^2$) | |
|---|---|---|---|
| No. of patients | Drug | Range | Median |
| 29 | Cisplatin | 170–607 | 400 |
| 15 | Ifosfamide | 10 200–96 000 | 20 000 |
| 12 | Vincristine | 4.7–16 | 9.4 |
| 10 | Methotrexate | 13 000–65 000 | 60 000 |
| 9 | Cisplatin<br>+<br>ifosfamide | 170–583<br>+<br>10 200–60 000 | 230<br>+<br>11 200 |
| 6 | Cisplatin<br>+<br>vincristine | 338–582<br>+<br>4.7–15 | 400<br>+<br>14 |

end of the great toe so that the weight of the probe (300 gm) was supported by the toe. The stimulus was increased from zero until the subject indicated having felt it, and the lowest of three readings was taken.[10] Cutaneous thresholds for warming and cooling were recorded from the plantar surface of the foot using a standard portable system as described by Fowler *et al.*[11] Criteria for overall abnormal nerve conduction studies suggestive of a sensory axonal neuropathy were defined as follows: (1) absent sural SNAP (sensory nerve action potential); or (2) abnormal VPT with either one of the following: (a) present but abnormal sural SNAP; or (b) absent medial plantar SNAP.

### *Autonomic function tests*

Sympathetic function was measured by postural changes in blood pressure. The subject lay supine for 20 min before the BP was recorded at 1 min intervals × 3. On standing, BP was recorded every minute for 5 min and then at 10 min. In normal subjects the systolic blood pressure does not fall > 15 mmHg.

Parasympathetic function was assessed by heart rate response to deep breathing and the Valsalva manouevre. The heart rate response to deep breathing was assessed using a continuous ECG during six full respiratory cycles over 1 min. The Valsalva maneouvre was used to evaluate parasympathetic function by asking the patient to maintain a forced expiratory pressure of 40 mmHg against an anaeroid pressure monitor dial.[12] The heart rate ratio was assessed and recorded three times; a normal ratio is $> 1.2$. The results were analysed using the values recommended by Ewing and Clarke.[12]

*Audiometry*

Hearing was assessed by bilateral audiograms and thresholds (dB) for detection were recorded between 250–12 000 Hz.

*Statistical analysis*

The data were analysed using the Statview statistical package on an Apple Macintosh computer. Two groups were compared using Student's *t*-test (continuous variables) and the chi-squared test (discrete variables). Significant correlations with age were found for the SNAP amplitudes and the VPTs. Significant correlation for height was found for the f-wave latency. Therefore, *t*-tests involving these variables were calculated after correction for age or height where relevant. All correlations were determined by calculating Kendall's rank correlation coefficient (tau).

### Results

*Clinical parameters and audiograms*

The only clinical neurological abnormality reported was Lhermitte's sign, which was reported by one patient and occurred during chemotherapy. There were no reports of symptomatic peripheral sensory or motor neuropathy, symptoms due to autonomic dysfunction, Raynaud's phenomenon, tinnitus or high tone hearing loss, as acute, intermediate or delayed effects. Clinical neurological assessment revealed no abnormalities of power (MRC scales), tone, or coordination, and no abnormalities of the clinically assessed sensory parameters, including joint position sense and vibration sense. However, the strikingly abnormal clinical parameter was the cumulative reflex score which was reduced to $\leq 6$ in 16/36 (44%) of patients. Bilateral audiograms were abnormal in 5/28 patients with minimal loss of high tone hearing. All five patients had received cisplatin (348–607 mg/m$^2$; median, 473 mg/m$^2$).

*Autonomic function tests*

Autonomic function tests were mildly abnormal in 4/22 patients tested. Two patients had minimal sympathetic abnormalities with systolic BP falling by 20 mmHg on standing. There was evidence of mild parasympathetic abnormality in two patients with an abnormal Valsalva ratio (1.2), and abnormal response to deep breathing (heart rate increase $< 15$ beats/min) in one patient. All four patients with mild autonomic dysfunction had received cisplatin (200–582 mg/m$^2$); three of these patients also had abnormal VPTs, and two had abnormal nerve conduction studies (NCS) suggestive of a sensory axonal neuropathy.

*Neurophysiology*

Normal limits for the neurophysiological parameters were determined in healthy volunteers and expressed as mean $\pm$ 2SD, except in the cases of medial plantar and sural SNAPs and VPT where the means and SDs of the log-transformed data were calculated and then converted back to original units to be used as limits of normal.[13,14] Results of all the neurophysiological data are shown in Table 4.

*Comparison of control group ($n = 20$) with patients who received cisplatin alone ($n = 15$).* These two groups were compared in the first instance because the patient group received only one neurotoxic drug (cisplatin) rather than several. The two groups were matched in age and height. The 15 patients differed significantly from the controls in the conduction study parameters for the two sensory nerves studied (medial plantar and sural), particularly the readings of the sensory nerve action potential amplitudes ($p < 0.0001$), but also the sensory conduction velocities ($p < 0.005$). There were also significant differences between the patient and control groups for vibration perception thresholds (VPTs) ($p < 0.001$), and f-wave latency ($p < 0.005$). No significant differences were observed for other motor parameters or thermal thresholds for warming and cooling.

An analysis of the correlation between cisplatin dose received (mg/m$^2$) and the neurophysiological parameters was made for these 15 patients. Vibration perception thresholds were significantly correlated with cisplatin dose received, but none of the other parameters measured.

*Analysis of all 29 patients who received cisplatin.* All neurophysiology parameters measured were analysed for possible correlation with the continuous variable of received cumulative dose of cisplatin (mg/m$^2$). The *only* neurophysiological parameter that was significantly correlated with dose of cisplatin in these patients was VPT (Fig. 1, $r = 0.607$, $p < 0.001$). The cumulative cisplatin dose (above or below 300 mg/m$^2$) also correlated with a normal or abnormal VPT (Fisher's exact test, $p = 0.005$) in this group of patients. Ten out of 29 (35%) patients had abnormal nerve conduction studies, showing a sensory axonal neuropathy. The presence of this

**Table 4.** *Nerve conduction studies: controls compared with patients receiving cisplatin alone*

| | Controls ($n = 20$) Mean ± SD | Normal limits | Patients ($n = 15$) Mean ± SD | $p$ (2-tail) | Cor/plat $p$ value |
|---|---|---|---|---|---|
| Age (years) | 22.0 ± 2.5 | | 20.8 ± 4.7 | 0.32 | |
| Height (cm) | 168 ± 8.9 | | 168 ± 6.8 | 0.89 | |
| Sensory tests | | | | | |
| mp SNAP* | 7.6 ± 3.7 | 2.4 μV | 2.3 ± 2.0 | < 0.0005 | 0.28 |
| mp DSL | 2.7 ± 0.44 | 3.6 ms | 3.4 ± 0.94 | < 0.01 | 1.0 |
| mp SCV | 45 ± 5.1 | 35 m s$^{-1}$ | 37 ± 7.5 | < 0.001 | 0.48 |
| surSNAP* | 16.6 ± 5.9 | 6.0 μV | 8.1 ± 4.5 | < 0.0005 | 0.37 |
| sur DSL | 3.1 ± 0.61 | 4.3 ms | 3.7 ± 0.68 | < 0.01 | 0.50 |
| sur SCV | 46 ± 4.9 | 36 m s$^{-1}$ | 40 ± 4.2 | < 0.0005 | 0.22 |
| Motor tests | | | | | |
| CMAP | 18.5 ± 6.6 | 5.3 mV | 15.0 ± 5.1 | 0.06 | 0.74 |
| DML | 3.7 ± 0.82 | 5.4 m s$^{-1}$ | 3.9 ± 0.58 | 0.51 | 0.11 |
| MCV | 47 ± 3.1 | 41 m s$^{-1}$ | 44 ± 4.8 | 0.07 | 0.47 |
| f-wave** | 47.0 ± 4.0 | | 50.0 ± 3.7 | < 0.005 | 0.87 |
| Vibration tests | | | | | |
| RVPT* | 5.3 ± 1.5 | 9 units | 12.1 ± 7.8 | < 0.005 | < 0.005 |
| LVPT* | 5.3 ± 1.2 | 8 units | 11.8 ± 7.6 | < 0.005 | < 0.05 |
| VPTsum* | 10.6 ± 2.5 | 17 units | 24.5 ± 15 | < 0.001 | < 0.05 |
| Thermal tests | | | | | |
| Cool TT | 0.31 ± 0.3 | 0.9°C | 0.48 ± 0.3 | 0.07 | 0.77 |
| Warm TT | 0.69 ± 0.6 | 1.9°C | 0.69 ± 0.6 | 0.99 | 0.10 |

SNAP, sensory nerve action potential; DSL, distal sensory latency; SCV, sensory conduction velocity; CMAP, compound muscle action potential; DML, distal motor latency; MCV, motor conduction velocity; VPT, vibration perception threshold; VPT sum, Sum of R and L VPT; TT, thermal threshold; mp, medial plantar nerve; sur, sural nerve; Cor/plat, correlation with cisplatin dose (mg m$^{-2}$), *After log transformation, **after correction for height.

neuropathy correlated with the cumulative total dose of cisplatin (mg/m$^2$) and reflex score, but showed no correlation with the treatment-free interval, or the dose of vincristine received (Table 5). None of the seven patients who received less than 300 mg/m$^2$ of cisplatin had neurophysiological evidence of sensory axonal neuropathy. There was also a significant correlation between reflex score and total received cisplatin dose ($r = -0.483$, $p = 0.009$).

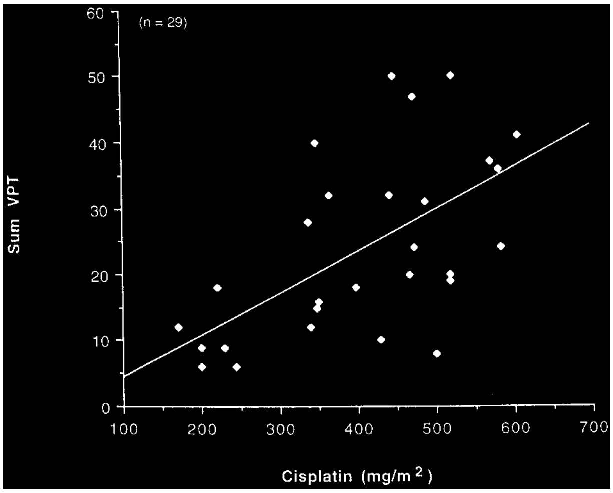

**Fig. 1.** *Total dose of cisplatin (mg/m$^2$) received versus sum VPT (vibration perception threshold) in 29 patients who received cisplatin ($r = 0.607$, $p < 0.001$).*

### *Correlation of reflex score with NCS abnormalities*

The only consistently abnormal clinical parameter in this group of adolescents and young adults was the reflex score. However this did prove useful as a simple clinical marker of an otherwise sub-clinical neuropathy. There was a significant correlation between reflex score and VPT (Fig. 2, $r = -0.548$, $p < 0.001$), medial plantar and sural SNAP, and medial plantar SCV (Table 6). Reflex score (normal or abnormal) correlated with both abnormal NCS ($p < 0.005$, Table 5) and abnormal VPT ($p < 0.005$).

### Discussion

Cisplatin is widely used in the treatment of bone sarcomas, but does produce well-recognised neurotoxicity which can limit its use.[15–17] Factors influencing the degree of neurotoxicity from cisplatin include total cumulative dose[15,18,19] the dose of each individual injection, the schedule of drug delivery[18,20–22] renal function, and coincident or previous neurological damage from other conditions.[23,24] Symptoms of peripheral parasthesiae characteristically can occur after cumulative doses of

**Table 5.** *Neurophysiological evidence of sensory axonal neuropathy*

| | No neuropathy (mean ± SD) | Neuropathy (mean ± SD) | $p$ (2-tail) |
|---|---|---|---|
| Age (years) | 20.4 ± 5 | 21.9 ± 5.0 | 0.42 |
| Height (cm) | 168.2 ± 12 | 172.0 ± 8.3 | 0.36 |
| TFI (weeks) | 65.6 ± 53.4 | 58.8 ± 46.2 | 0.77 |
| Cisplatin (mg m $^{-2}$) | 342.2 ± 129 | 472.1 ± 83.3 | < 0.01* |
| Vincristine (mg) | 19.2 ± 8.7 | 10.7 ± 1.2 | 0.13 |
| Reflex score | 7.8 ± 2.5 | 4.0 ± 3.4 | < 0.005* |

TFI, treatment-free interval in weeks.
*Statistically significant result.

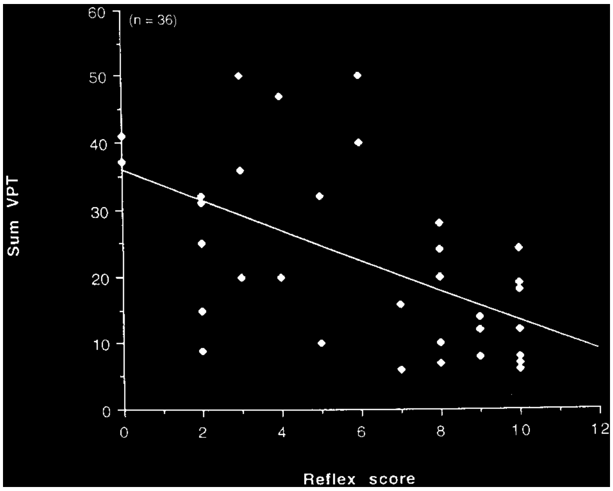

**Fig. 2.** *Reflex score versus sum VPT (vibration perception threshold) in all 36 patients studied ($r = -0.548$, $p < 0.001$).*

300 mg/m$^2$ or greater, and at doses greater than 600 mg/m$^2$ severe disabling sensory ataxia and motor problems may be seen. Increases in VPT are often the earliest manifestation of peripheral neuropathy[25] usually preceding symptoms and signs by 6–8 weeks.[26] They are found to be significantly elevated in patients who have received doses of cisplatin > 300 mg/m$^2$.[27] This is followed by a reduction in amplitude[4] or absence[28] of sensory nerve action potentials, consistent with a sensory axonal neuropathy or ganglioneuropathy. It appears that the neuropathy is due to a toxic effect on the dorsal root ganglia, where tissue platinum levels have been found to be the highest.[18,29] These sensory ganglia are relatively poorly protected by the blood barrier and, as such, seem to be the most vulnerable neural structures to the effects of neurotoxins.[30,31] Marked degeneration and gliosis of the dorsal columns with significant axonal loss of the dorsal roots were reported in a 3-year-old girl who had received 960 mg/m$^2$ of cisplatin.[32] The dorsal column involvement is thought to be responsible for the occasional case of Lhermitte's sign reported in association with the neuropathy.[33,34] There is still some uncertainty whether the major cisplatin-induced damage to the human sensory peripheral nerves is at the level of the cell body, the axon, or both. Although cisplatin toxicity is known to involve predominantly large-diameter sensory fibres, an autonomic neuropathy has been described[14,35] occurring in patients receiving a combination of cisplatin, vinblastine and bleomycin, and this has been postulated to be a 'dying back' small-fibre neuropathy. A motor component to the neuropathy has been reported to occur in some patients receiving high doses of cisplatin.[36]

This report adds to the experience of cisplatin neuropathy in young people, and provides a comparison with an age-matched control population. We have found evidence of a persisting sensory axonal neuropathy in this group of young people who received cisplatin as part of adjuvant chemotherapy treatment for bone and soft tissue sarcomas. Ten of 29 patients (33%) who received cisplatin had evidence of a sensory axonal neuropathy at a median of 8 months after the completion of chemotherapy. This is manifest by a reduction in the amplitude of the sensory action potentials in sural and medial

**Table 6.** *Correlations of neurophysiological parameters with reflex score ($n = 36$)*

| | Linear Pearson's $r$ | Rank Kendall's $t$ | $p$ value |
|---|---|---|---|
| Medial plantar SNAP | 0.52 | 0.37 | < 0.005* |
| Medial plantar SCV | 0.66 | 0.33 | < 0.05* |
| Sural SNAP | 0.50 | 0.39 | < 0.05* |
| RVPT | − 0.53 | − 0.36 | < 0.005* |
| LVPT | − 0.52 | − 0.34 | < 0.01* |
| VPT SUM | − 0.55 | − 0.38 | < 0.05* |
| RVPT/AGE | − 0.60 | − 0.37 | < 0.005* |

*Statistically significant result.

plantar nerves, and an increase in the VPT. Interestingly the only clinical indication of neurological damage was transient Lhermitte's sign in one patient which persisted for only 2 months during chemotherapy. No patients had parasthesiae, or symptomatic sensory loss during chemotherapy or as an immediate or late effect. Despite this nearly one-third of patients were demonstrated to have a sensory axonal neuropathy on NCS. The only clinically detected abnormality was a reduction in reflex score, which was seen to a significant extent in 16/36 (44%) of patients. The reflex score correlated with neurophysiological measurements, showing significant correlations with VPT, amplitude of SNAP and SCV. Audiometric abnormalities were demonstrated in 5/28 patients but these changes were minor, and no patient had tinnitus or symptomatic hearing loss. Minor abnormalities of autonomic function were demonstrated in 4/22 patients, and whether these patients are at significant risk from cardiorespiratory arrest, which has been reported in patients with diabetes and autonomic dysfunction,[37] is uncertain, but seems unlikely.

Of some interest is the finding of significant prolongation of f-wave latencies in the patient group as a whole, which persisted after controlling for height, and the relative slowing in motor conduction in the posterior tibial nerve. As there is no sensory component to the f-wave latency measurement, this suggests that subtle motor involvement is detectable, even in mild asymptomatic cases such as those reported here. This could be a result of either a mild diffuse disorder of myelination causing a slight slowing of conduction velocity, or it could be the result of mild axonal loss in which the large fastest conducting fibres are lost first, resulting in a change in the minimum f-wave latency, but not sufficient to cause a noticeable decrease in the CMAP amplitude. The degree of axonal loss could be assessed by further EMG studies of such a group of patients.

The VPT emerged in this study as the most valuable single neurophysiological test in the assessment of cisplatin-induced neuropathy in our patients. Measurement of VPT using the Biothesiometer is a simple technique which does not require the experienced training necessary for the carrying out of other more sophisticated nerve conduction studies. The Biothesiometer is cheap, and easily portable, and the test itself takes less than a minute to perform and is entirely painless for the patient. It is a quantitative measure which significantly correlated with cumulative cisplatin dose, and could also be of use particularly in following recovery after completion of chemotherapy. In further research into cisplatin peripheral neuropathy, VPT measurement using a Biothesiometer could be recommended. This will be particularly useful for long-term follow-up of younger patients. Developments in this area of clinical cancer research are likely to include the development of neuroprotectors, and the ease and acceptability of VPT measurement with the Biothesiometer would make this an obvious choice for collection of repeated measurements within the context of randomised clinical trials.

Reports concerning the progress of the neuropathy are anecdotal. Improvement of the neuropathy over time has been reported by some authors but not by others.[25] It seems that some improvement may occur after cessation of therapy, but deficits do remain affecting vibratory perception and proprioception, which are considered largely irreversible[38] and which may progress.[39–41] In our study there was no correlation between the time from chemotherapy and any of the clinical or neurophysiological parameters, and this would suggest that there is not significant recovery, through time, of the subclinical peripheral neuropathy documented in these young people.

Cisplatin is used in combination with other cytotoxics in a number of chemo-curable malignancies which affect young people, including bone sarcomas and germ cell tumours. There has been recent concern that its substitution by the less neurotoxic analogue carboplatin in the treatment of metastatic germ cell tumours has led to a significant worsening in outcome, because of a possible reduction in efficacy.[42] It is likely that, for the foreseeable future, cisplatin will remain an important component of combination chemotherapy in a number of malignancies in adolescents and young adults. Our study adds further to the evidence of substantial neurotoxicity of cisplatin in this group, in comparison with age-matched controls, and lends important evidence to arguments for the development of effective neuroprotectors.[21,43] The precise molecular mechanisms of the neurological damage produced by cisplatin are unknown. Possibilities include the reaction of cisplatin with DNA in the dorsal root ganglia to reduce the production of nerve growth factor, or other neurotrophic proteins responsible for the maintenance of an intact peripheral nervous system. Other possibilities include the reaction of cisplatin with neurotransport proteins. Whatever the mechanisms of toxicity, the development of neuroprotectors will be increasingly important. Several agents have already been developed and used in this role.[44] These include WR 2721, a thiol derivative, which has been used with the aim of reducing haematological, renal and neurotoxicity.[39] Org 2766, a four-amino acid derivative of ACTH[45] has been used successfully in the prevention and amelioration of neurotoxicity in patients with ovarian cancer.[46] Exciting developments have recently been reported, with the newly recognised nerve growth factors in the regeneration of the peripheral nervous system[47] and the prevention of experimental cisplatin neuropathy.[48] Insulin-like growth factor-I (IGF-I)[49] has also been found to be a potent neurotrophic agent in *in vivo* testing in an experimental rat model and is entering Phase 2 testing in degenerative neurological diseases. However IGF-I's role as an active growth factor in many tumours has delayed its testing in cancer patients until the results of further *in vitro* and *in vivo* tests are available.

### References

1 Stiller CA, Bunch KJ. Trends in survival for childhood cancer in Britain diagnosed 1971–85. *Br J Cancer* 1990; 62:806–15.
2 Craft A. Chemotherapy of Ewing's Sarcoma. In: Souhami RL, ed. *Bone Tumours*. London, Philadelphia, Toronto, Sydney, Tokyo: Bailliere Tindall, 1987: 205–21.
3 Boyer M, Raghavan D, Harris PJ, *et al.* Lack of late toxicity in patients with cisplatin-containing combination chemotherapy for metastatic testicular cancer. *J Clin Oncol* 1990; 8:21–6.
4 Daugaard GK, Petrera J, Trojaborg W. Electrophysiological study of the peripheral and central neurotoxic effect of cisplatin. *Acta Neurol Scand* 1987; 76:86–93.
5 Rosen G, Marcove RC, Huvos AG, *et al.* Primary osteogenic sarcoma: eight years experience with adjuvant chemotherapy. *J Cancer Res Clin Oncol* 1983; 106(Suppl): 55–67.
6 Rosen GA. Preoperative (neoadjuvant) chemotherapy for osteogenic sarcoma: a ten year experience. *Orthopedics* 1985; 8:659–64.
7 Earl HM, Pringle J, Kemp H, *et al.* Chemotherapy of malignant fibrous histiocytoma of bone. *Ann Oncol* 1993; 4:409–15.
8 Heier MS, Nilsen T, Graver V, *et al.* Raynaud's phenomenon after combination chemotherapy of testicular cancer measured by laser doppler flowmetry. A pilot study. *Br J Cancer* 1991; 63:550–2.
9 Fossa SD, Aass N, Ous S, *et al.* Long-term morbidity and quality of life in testicular cancer patients. *Scand J Urol Nephrol* 1991; 138:241–6.
10 Nielsen VK. The peripheral nerve function in chronic renal failure, IV. An analysis of the vibration perception threshold. *Acta Med Scand* 1972; 191:287–96.
11 Fowler CJ, Carroll MB, Burns D, *et al.* A portable system for measuring cutaneous thresholds for warming and cooling. *J Neurol Neurosurg Psychiatry* 1987; 50:1211–5.
12 Ewing DJ, Clarke B. Autonomic neuropathy: its diagnosis and prognosis. *Clin Endocrinol Metab* 1986; 15:855–88.
13 Robinson R. Effect of statistical methodology on normal limits in nerve conduction studies. *Muscle Nerve* 1991; 14:1084–90.
14 Robinson R, Cantwell BMJ. Autonomic neuropathy after cisplatin based chemotherapy. Br Med J 1990; 300:466–75.
15 Cersosimo RJ. Cisplatin neurotoxicity. *Cancer Treat Rev* 1989; 6:195–211.
16 van der Hoop RG, van der Burg ME, ten Bokkel-Huinink WW, *et al.* Incidence of neuropathy in 395 patients with ovarian cancer treated with or without cisplatin. *Cancer* 1990; 66:1697–702.
17 Boogerd W, ten Bokkel Huinink WW, Dalesio O, *et al.* Cisplatin induced neuropathy: central, peripheral and autonomic nerve involvement. *J Neurooncol* 1990; 9:255–63.
18 Gregg RW, Molepo JM, Monpetit VJ, *et al.* Cisplatin neurotoxicity; the relationship between dosage, time, and platinum concentration in neurologic tissues, and morphologic evidence of toxicity. *J Clin Oncol* 1992; 10:795–803.
19 Hakes T, Hoskins W, Jones W, *et al.* Randomised prospective trial of 5 versus 10 cycles of cyclophosphamide, doxorubicin, and cisplatin (CAP) in stage III and IV ovarian cancer. *Proc Am Soc Clin Oncol* 1990; 9:Abstr 606.
20 Sebille A, St-Guily JL, Angelard B, *et al.* Low prevalence of cisplatin-induced neuropathy after 4-day continuous infusion in head and neck cancer. *Cancer* 1990; 65:2644–7.
21 Gandara DR, Perez EA, Hoff J, *et al.* High dose cisplatin: modulation of toxicity. San Diego, CA: Sixth International Symposium on Platinum and other Metal Coordination Compounds in Cancer. 1991: 51.
22 Hainsworth JD, Burnett LS, Jones HW, *et al.* High dose cisplatin combination chemotherapy in the treatment of advanced epithelial ovarian cancer. *J Clin Oncol* 1990; 8:502–8.
23 Cavaletti G, Petruccioli MG, Tredici G, *et al.* Effects of repeated administration of low dose cisplatin on the rat nervous system. *Int J Tissue React*, 1991; 13:151–7.
24 Kim HK, Lee KS, Hong YS, *et al.* Cisplatin-induced peripheral neuropathy in patients with a history of neurologic damage. *Proc Am Soc Clin Oncol* 1991; 10:Abstr 1224.
25 Ashraf M, Riggs JE, Wearden S, *et al.* Prospective study of nerve conduction parameters and serum magnesium following cisplatin therapy. *Gynaecol Oncol* 1990; 37:29–33.
26 Thompson SW, Davis LE, Kornfeld M, *et al.* Cisplatin neuropathy. Clinical, electrophysiologic, morphologic, and toxicologic study. *Cancer* 1984; 54: 1269–75.
27 Elderson A, van der Hoop RG, Haanstra W, *et al.* Vibration perception and thermoperception as quantitative measurements in the monitoring of cisplatin induced neurotoxicity. *J Neurol Sci* 1989; 93:167–74.
28 Cowan JD, Kies MS, Roth JL, *et al.* Nerve conduction studies in patients treated with cis-diamminedichloroplatinum (II): a preliminary report. *Cancer Treat Rep* 1980; 64:1119–22.
29 Hansen SW, Helweg-Larson S, Trojaborg W. Longterm neurotoxicity in patients treated with cisplatin, vinblastine, and bleomycin for metastatic germ cell cancer. *J Clin Oncol* 1989; 7:1457–61.
30 Cavanagh JB. Sensorimotor neuropathy and cisplatin and adriamycin toxicity. *J Neurol Neurosurg Psychiatry* 1986; 49:964–5.
31 Tomiwa K, Nolan C, Cavanagh JB. The effects of cisplatin on rat spinal ganglia. A study by light and electron microscopy and by morphometry. *Acta Neuropathol* 1986; 69:295–308.
32 Walsh TJ, Clark AW, Parhad IM, *et al.* Neurotoxic effects of cisplatin therapy. *Arch Neurol* 1982; 39:719–20.
33 Dewar J, Lunt H, Abernethy DA, *et al.* Cisplatin neuropathy with Lhermitte's sign. *J Neurol Neurosurg Psychiatry* 1986; 49:96–9.
34 List AF, Kummet KD. Spinal cord toxicity complicating treatment with cisplatin and etoposide. *Am J Clin Oncol* 1990; 13:256–8.
35 Hansen SW. Autonomic neuropathy after treatment with cisplatin, vinblastine, and bleomycin for germ cell tumours. Br Med J 1990; 300:511–2.
36 Pages M, Pages AM, Bories-Azeau L. Severe sensorimotor neuropathy after cisplatin therapy. *J Neurol Neurosurg Psychiatry* 1986; 49:333–4.
37 Samuels BL, Vogelzang NJ, Kennedy BJ. Severe vascular toxicity associated with vinblastine, bleomycin and cisplatin chemotherapy. *Cancer Chemother Pharmacol* 1987; 19:253–6.
38 Mollman JE. Cisplatin Neuropathy. *New Engl J Med* 1990; 322:126–7.

39 Mollman JE, Glover DJ, Hogan WM, *et al.* Cisplatin neuropathy risk factors, prognosis, and protection by WR-2721. *Cancer* 1988; 61:2192–5.
40 Siegal T, Haim N. Cisplatin-induced peripheral neuropathy. Frequent off-therapy deterioration, demyelinating syndromes, and muscle cramps. *Cancer* 1990; 66: 1117–23.
41 Grunberg SM, Sonka S, Stevenson LL, *et al.* Progressive parasthesias after cessation of therapy with very high dose cisplatin. *Cancer Chemother Pharmacol* 1989; 25:62–4.
42 Horwich A, Sleijfer D, Fossa S, *et al.* A trial of carboplatin-based chemotherapy in good prognosis metastatic testicular non-seminoma. *Proc Soc Clin Oncol* 1994; 13:Abstr 709.
43 Hamers FP, van der Hoop RG, Steerenburg PA, *et al.* Putative neurotrophic factors in the protection of cisplatin induced peripheral neuropathy in rats. *Toxicol Appl Pharmacol* 1991; 111:514–22.
44 Pirovano C, Bohm S, di Re F, *et al.* Clinical and neurophysiological evaluation of peripheral neurotoxicity following high-dose cisplatin with glutathione protection in the treatment of ovarian cancer. *Anticancer Res* 1990; 10:1426.
45 Muller LJ, Van der Hoop RG, van Delft MCM, *et al.* Morphological and electrophysiological study of the effects of cisplatin and ORG 2766 on rat spinal ganglion neurons. *Cancer Res* 1990; 50:2437–42.
46 van der Hoop RG, Vecht CJ, van der Berg MEL, *et al.* Prevention of cisplatin neurotoxicity with an ACTH (4–9) analogue in patients with ovarian cancer. *New Engl J Med* 1990; 322:89–94.
47 Raivich G, Kreutzberg G. Nerve growth factor and regeneration of the peripheral nervous system. *Clin Neurol Neurosurg* 1993; 95 (Suppl):S84–8.
48 Apfel SC, Arezzo JC, Lipson L, *et al.* Nerve growth factor prevents experimental cisplatin neuropathy. *Ann Neurol* 1992; 31: 76–80.
49 Sara VR. The role of insulin-like growth factors in the nervous system. In: Schofield PN, ed. *The Insulin-Like Growth Factors. Structure and Biological Functions.* Oxford, New York, Tokyo: Oxford University Press, 1992:258–79.
